# PGC-1*α*-Mediated Mitochondrial Quality Control: Molecular Mechanisms and Implications for Heart Failure

**DOI:** 10.3389/fcell.2022.871357

**Published:** 2022-05-27

**Authors:** Lei Chen, Yuan Qin, Bilin Liu, Meng Gao, Anqi Li, Xue Li, Guohua Gong

**Affiliations:** ^1^ Institute for Regenerative Medicine, Shanghai East Hospital, School of Life Sciences and Technology, Tongji University, Shanghai, China; ^2^ Department of Pharmacy, Shanghai East Hospital, Tongji University, Shanghai, China

**Keywords:** PGC-1*α*, mitochondrial biogenesis, mitochondrial quality control, heart failure, mitochondrial dynamics

## Abstract

Mitochondria with structural and functional integrity are essential for maintaining mitochondrial function and cardiac homeostasis. It is involved in the pathogenesis of many diseases. Peroxisome proliferator-activated receptor *γ* coactivator 1 *α* (PGC-1*α*), acted as a transcriptional cofactor, is abundant in the heart, which modulates mitochondrial biogenesis and mitochondrial dynamics and mitophagy to sustain a steady-state of mitochondria. Cumulative evidence suggests that dysregulation of PGC-1*α* is closely related to the onset and progression of heart failure. PGC-1*α* deficient-mice can lead to worse cardiac function under pressure overload compared to sham. Here, this review mainly focuses on what is known about its regulation in mitochondrial functions, as well as its crucial role in heart failure.

## Introduction

Mitochondria comprise ∼40% of the volume of myocytes and produce ∼95% of the ATP (Liu et al., 2010; Riehle and Abel, 2012; Zhou and Tian, 2018). The number, morphology, and function of mitochondria are well maintained under normal physiological conditions, which keeps the cardiac homeostasis. It is widely recognized that mitochondrial quality control (MQC) system is essential for maintaining a healthy and functional mitochondrial network. MQC is a complex network that involves specific removal of damaged mitochondria, the supplement of fresh mitochondria by mitochondrial biogenesis, the separation of damaged mitochondria by fission, and the exchange of mitochondrial content by fusion (Gottlieb et al., 2021). Alteration of these processes can lead to mitochondrial dysfunction. The dysfunctional mitochondria have closely associated with heart failure (HF).

**FIGURE 1:**
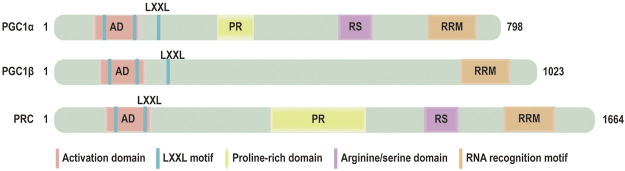
Domain structure of PGC‐1 coactivators: the functional domains of PGC‐1 coactivators: activation domain (AD), LXXL motif, a proline-rich domain, arginine/serine (RS) domain, RNA recognition motif (RRM).

Peroxisome proliferator-activated receptor *γ* coactivator 1 *α* (PGC-1*α*) is originally identified as a coactivator of nuclear receptors in brown fat (Puigserver et al., 1998). PGC-1*α* belongs to a small family of transcriptional coactivators, which composes the other two members: Peroxisome proliferator-activated receptor *γ* coactivator 1 *β* (PGC-1*β*) and PGC-1 related coactivator (PRC) (Puigserver and Spiegelman, 2003) (Figure 1). It has been proved that PGC-1a lacks intrinsic enzymatic activity or DNA binding domain. However, PGC-1*α* is interacted with transcription factors nuclear respiratory factor 1 and 2 (NRF1/NRF2) and mitochondrial transcription factor A (TFAM) to modulate mitochondrial biogenesis and mitochondrial energy metabolism Beyond this function, PGC-1*α* also plays an important role in mitochondrial dynamics and mitophagy *via* modulating the pivotal factors of these processes, including mitofusin2 (MFN2), dynamin-related protein 1 (DRP1), PTEN-induced putative kinase protein1 (PINK1) and PARKIN. PGC-1*α* is subject to both the transcriptional regulation and posttranslational modifications that alter its activity and expression. The change of PGC-1*α* is relevant to the development and progression of HF. Some studies have shown that the expression of PGC-1*α* is decreased in the advanced stage of heart failure accompanied by impairment of mitochondrial number, structure, function (Lehman and Kelly, 2002; Arany et al., 2006). Recent studies illuminate that PGC-1*α* expression is varied in HF (Hu et al., 2008; Wang et al., 2018; Bhat et al., 2019).

In this review, we focus on the current understanding of its regulation of the mitochondrial network and its role in MQC, energy metabolism and heart failure.

## PGC-1a and its Regulation of Mitochondrial Energy Metabolism

Mitochondria are cellular powerhouse, which generate ATP *via* mitochondrial respiratory chain. Acetyl-coenzyme A (CoA), produced from fatty acids, amino acids or pyruvate oxidation, triggered the initiation of tricarboxylic acid cycle (TCA) cycle, during which NAD^+^ and FAD are convert to NADH and FAD (Martinez-Reyes and Chandel, 2020). Electrons from NADH and FADH are transferred to complexes of electron transport chain (ETC), which ultimately contributed to proton transport across the mitochondrial inner membrane to drive ATP synthesis (Martinez-Reyes and Chandel, 2020).

Heart has extremely energy demand and 70% of its energy comes from Oxidation of fatty acids (FAs). PGC-1*α* is highly expressed in the heart, which maintains fatty acid oxidation rates and mitochondrial respiratory function (Huss and Kelly, 2004). The PGC-1*α* increased at birth accompanied by an increase in cardiac oxidative capacity and a shift metabolism from glycolysis to oxidative phosphorylation (Lehman et al., 2000). Accumulating evidence displays that PGC-1*α* is involved in regulation of cardiac energy metabolism *via* interacting with three important transcription factors. First, PGC-1*α* interacts with peroxisome proliferator-activated receptor *α* (PPAR*α*), modulating expression of enzymes involved in fatty acid uptake and mitochondrial fatty acid oxidation (Vega et al., 2000; Panagia et al., 2005). Moreover, estrogen receptor related receptor*α* (ERR*α*) is an orphan nuclear receptor that is activated by PGC-1*α* in the myocardium, promoting increase of expression of (fatty acids oxidation) FAO and OXPHOS enzymes (Schreiber et al., 2003). Finally, NRF1 serves as a downstream target of PGC-1*α* that regulates transcription of genes involved in mitochondrial OXPHOS and enhances the expression of mitochondrial complexes I, II, III, IV, and Cytochrome C (CytC) (Gleyzer et al., 2005).

PGC-1*α* is activated in stressful conditions (Fasting, cold, exercise) to meet demand of high energy (Huss and Kelly, 2005). Overexpression of PGC-1*α* in heart enhanced expression of metabolic regulators including TCA cycle enzyme (citrate synthase) and components of the oxidative phosphorylation complex and components of the electron transport chain involved (Lehman et al., 2000). Reduction of PGC-1*α* expression results in cardiac metabolic defect. It has reported that deletion of PGC-1*α* in mice contributes to a 30∼50% reduction of genes (*Cycs*, *Cox5b*, *Atp5o*, *Ndufb5*, *Mcad*, *Cpt1*, *Cpt2*) expression involved in oxidative phosphorylation, fatty acid oxidation and ATP synthesis (Arany et al., 2005). Similarly, Chang and colleagues detect and analyze cardiomyocyte energy metabolism profile in heart specific PGC-1*α* deletion mice. The result shows that acetylation of both CoA and L-carnitine was suppressed suggesting that production of acetyl groups from oxidation of both carbohydrates and fatty acids are reduced (Karkkainen et al., 2019). Besides, reduction of succinic acid level in KO hearts repressed oxidative phosphorylation capacity. The levels of NAD^+^ and FAD are also decreased, further leading to the decreased capacity of ATP production in PGC-1*α* lacking mice. Chang group also analyzed correlation between metabolites alteration and cardiac function parameters. Result of the analysis illustrates that glycerophosphate and breakdown product of PCs, are associated directly with ejection fraction.

## PGC-1*α* and Its Regulation of Mitochondrial Quality Control

Mitochondrial quality control is defined as an extremely complex process including mitochondrial biogenesis (generating new mitochondria), dynamics (maintaining genetic and biochemical uniformity), mitophagy (removing damaged mitochondria). The coordination among these processes is essential for the maintenance of quantity, morphology, and function of mitochondria (Figure 5) (Wang et al., 2020). Under physiological conditions, mitochondrial biogenesis, mitochondrial dynamics, and mitophagy are precisely regulated, which further maintainsthe balance and stability of the MQC network (Figure 5). PGC-1*α*, as a critical MQC modulator, is involved in mitochondrial biogenesis, mitochondrial dynamics and mitophagy (Zhang et al., 2019).

### PGC-1*α* and its Regulation of Mitochondrial Biogenesis

**FIGURE 2:**
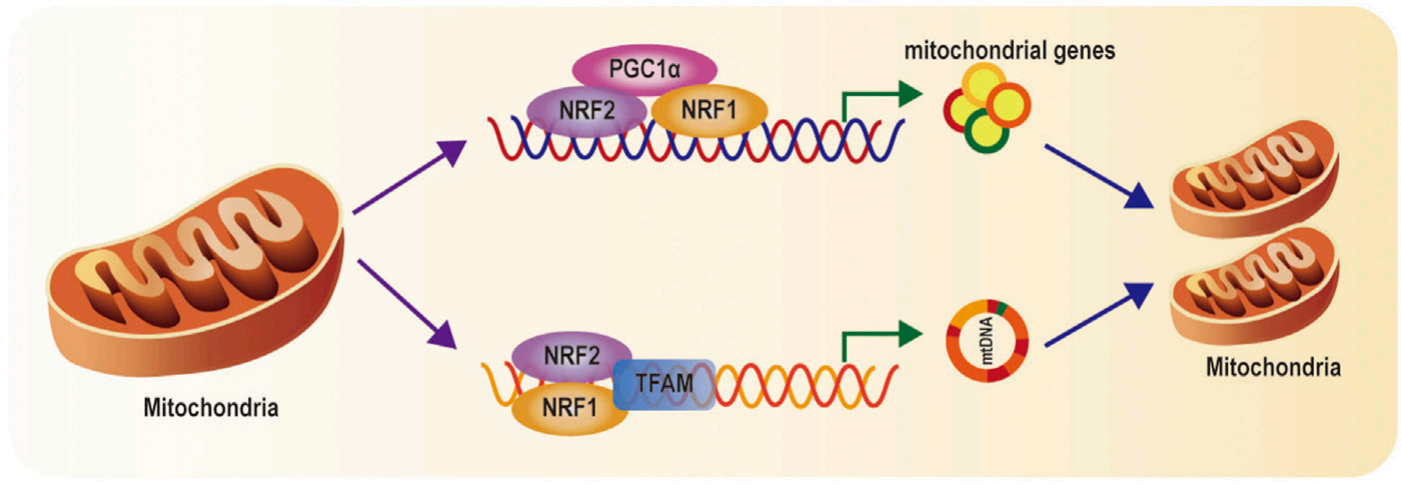
Mitochondrial biogenesis pathways: When PGC-1*α* is activated, PGC-1a activates NRF1 and NRF2, and subsequently TFAM, which regulate genes involved in subunits of mitochondrial respiratory chain complexes, import of nuclear-encoded mitochondrial proteins, and mtDNA replication and transcription.

Mitochondrial biogenesis implicates the generation of new healthy mitochondria to meet the requirement of biological energy and replenish damaged mitochondria (Popov, 2020). Mitochondrial biogenesis is an extremely intricate process including the synthesis of the mtDNA encoded proteins and the imports of nuclear encoded mitochondrial proteins and replication of mitochondrial DNA (mtDNA) (Li et al., 2017; Uittenbogaard and Chiaramello, 2014). Mitochondrial biogenesis is dependent on coordinated regulation of mitochondrial and nuclear factors (Ploumi et al., 2017). It has been reported that PGC-1*α* acts as a critical regulator of mitochondrial biogenesis *via* the transcriptional machinery to increase mitochondrial mass. Stressors (nutrient deprivation, hypoxia, oxidant stress, or exercise) activate PGC-1*α* activity and enhance its level, then inducing its location from the cytoplasm to the nucleus. Activated PGC-1*α* results in increase of NRF1 and NRF2 expression. Activation of NRF1 and NRF2 promotes the transcription of many mitochondrial genes involved in subunits mitochondrial respiratory chain complexes (Figure 2) (Wu et al., 1999). Simultaneously, NRF1 and NRF2 also stimulate the synthesis of TFAM, which subsequently mediates mtDNA replication and transcription (Figure 2) (Kelly and Scarpulla, 2004; Gleyzer et al., 2005; Vina et al., 2009). Finally, the PGC-1a—NRF1/2—TFAM pathway contributes to the formation of new mitochondria.

**FIGURE 3:**
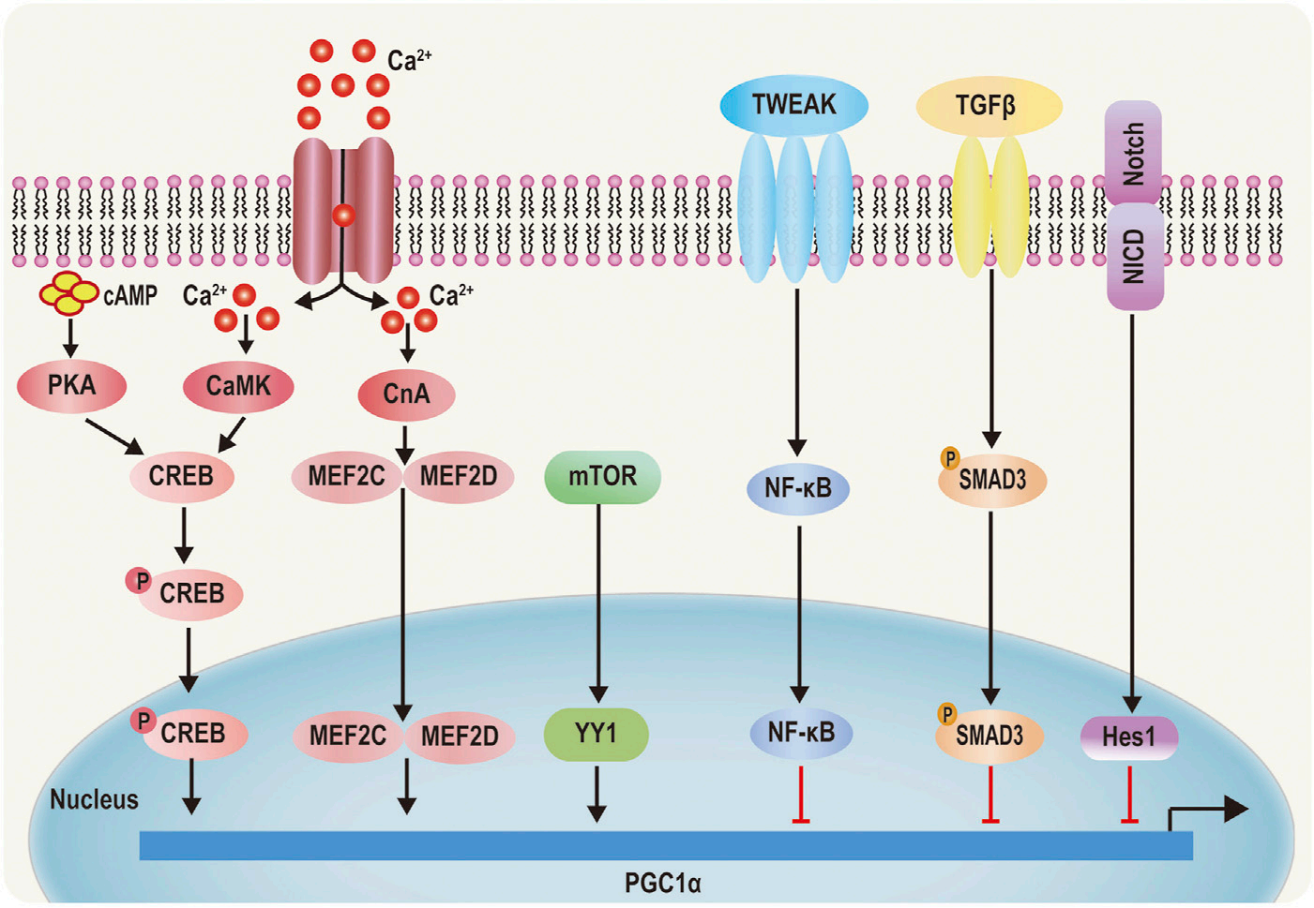
Transcriptional regulation of PGC‐1*α*. 1) PKA is activated by cyclic AMP (cAMP) and CaMKIV is activated by Ca^2+^ signaling phosphorylate CREB, which increases PGC‐1*α* activity. 2) Elevated cytoplasmic Ca^2+^ activates CnA, which regulates MEF2C and MEF2D, causing MEF2C and MEF2D to translocate into the nucleus and initiate PGC‐1*α* transcription. 3) mTOR‐induced YY1 increases PGC‐1*α* promoter activity. 4) TWEAK TGF‐*β* and Notch can inhibit PGC‐1*α* promoter activity by the _P_SMAD3, Rel A, NF‐kB, and Hes1 pathways, respectively.

PGC-1*α* expression and activity are modulated by transcriptional and posttranslational levels (Scarpulla, 2008). Transcriptional regulation is the central approach to increase the total expression and activity of PGC-1*α*. Increase of cyclic adenosine monophosphate (cAMP) concentration activates protein kinase A (PKA), mediating phosphorylation of cAMP-response element-binding protein (CREB) at Ser 133 (Herzig et al., 2001). Ca^2+^ is interacted with Ca^2+^/calmodulin‐dependent protein kinase (CaMK), which induces phosphorylation of CREB (Mattson, 2012) Phosphorylation of CREB eventually results in enhancement of PGC-1*α* level (Figure 3) (Uittenbogaard and Chiaramello, 2014). Ca^2+^ also triggers the activation of calcineurin A (CnA). once activated, CnA interacts with myocyte enhancer factors 2C and 2D (MEF2C and MEF2D) and strongly drives PGC-1*α* expression (Figure 3) (Handschin et al., 2003; Stotland and Gottlieb, 2015). Besides, Ca^2+^ can contribute to the activation of AMPK, which increases expression of PGC-1*α* (Choi et al., 2016). Mammalian target of rapamycin (mTOR) can modulate yin yang 1(YY1)–PGC-1*α* interaction, which then mediates increases of PGC-1*α* promoter activity (Cunningham et al., 2007). On the contrary, TWEAK, an inflammation factor, induces activation of nuclear factor-κB (NF-κB). Then, NF-κB translates to nuclear along with recruitment of histone deacetylase (HDAC), which subsequently reduces histone acetylation, suppressing PGC-1*α* expression (Ruiz-Andres et al., 2016; Shi et al., 2013). In addition, Hes1 directly binding the PGC-1*α* promoter region (a downstream target of fibrotic Notch signaling) decreases PGC-1*α* levels (Figure 3) (Han et al., 2017). Moreover, transforming growth factor*β* (TGF*β*)—induced phosphorylation of Smad3 directly binding to PGC-1*α* promoter represses PGC-1*α* expression (Figure 3) (Yadav et al., 2011).

**FIGURE 4:**
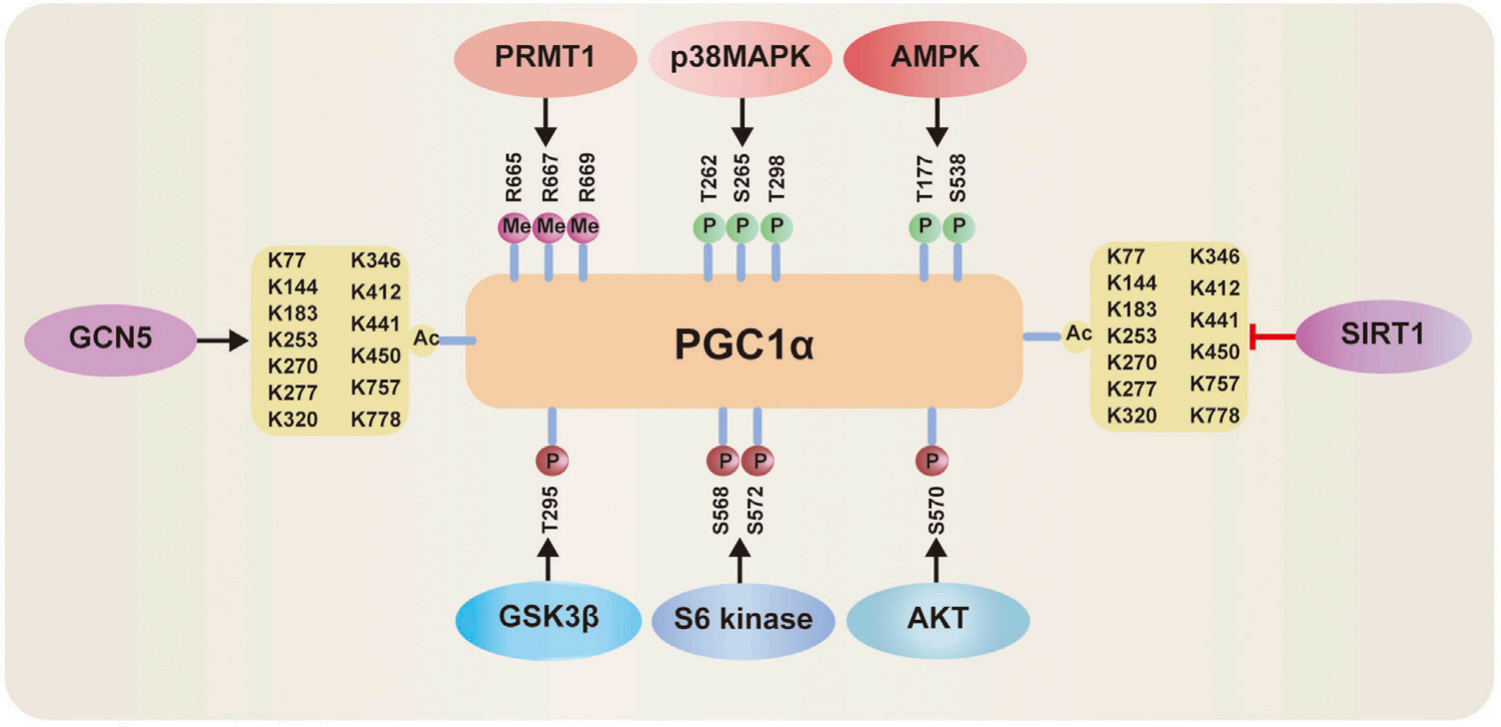
Posttranslational modifications of PGC‐1*α*: 1) PGC-1*α* activity can be activated *via* methylation by protein arginine methyltransferase1 (PRMT1), phosphorylated by p38 mitogen-activated protein kinase (MAPK) and AMP-activated protein kinase (AMPK), deacetylation by Sirtuin-1 (SIRT1). 2) PGC-1*α* activity can be inhibited *via* acetylation by general control of amino acid synthesis 5 (GCN5), phosphorylation by Akt, S6 Kinase, or glycogen synthase kinase 3*β* (GSK3*β*).

Posttranslational modifications including methylation, phosphorylation and deacetylation can regulate PGC-1*α* levels. PGC-1*α* is methylated by protein arginine methyltransferase1 (PRMT1) at arginine (Arg) 665, 667, and 669 (Figure 4), which induces the enhancement of PGC-1*α* activity, thus mediating the expression of essential target genes that are involved in mitochondrial biogenesis (Teyssier et al., 2005). Phosphorylated PGC-1*α* at threonine (Thr) 262, serine (Ser) 265, and Thr 298 by p38 mitogen-activated protein kinase (MAPK) (Figure 4) disrupts the interaction between PGC-1*α* and its inhibitor p160MBP, which increases its activity (Barger et al., 2001; Tang, 2016). Recent a study shows that inhibition of p38 MAPK markedly repressed the expression of PGC-1*α* (Ye et al., 2019). Activation of AMP-activated protein kinase (AMPK) induced by the elevated AMP/ATP ratio directly phosphorylates PGC-1*α* at Thr 177 and Ser 538 Jager et al., 2007. Furthermore, this phosphorylation can increase the occupancy of PGC-1*α* at the promoters of its target genes. In addition, AMPK increases nicotinamide adenine dinucleotide (NAD^+^) levels, thus enhancing Sirtuin1 (SIRT1) activity, which results in activation of PGC-1*α* by deacetylation (Figure 4) (Canto et al., 2009; Nemoto et al., 2005). Conversely, when NAD^+^ intracellular concentrations decrease, general control of amino acid synthesis 5 (GCN5) acetylates PGC-1*α* with a decrease in its transcriptional activation (Dominy et al., 2010; Kelly et al., 2009; Gerhart-Hines et al., 2007). Inhibition of PGC-1*α* can occur *via* phosphorylation by AKT at Ser 570 Li et al., 2007, S6 kinase at Ser 568 and Ser 572 Lustig et al., 2011, or glycogen synthase kinase 3*β* (GSK3*β*) at Thr 295 (Figure 4) (Anderson et al., 2008).

### PGC-1*α* and Mitochondrial Dynamics

**FIGURE 5:**
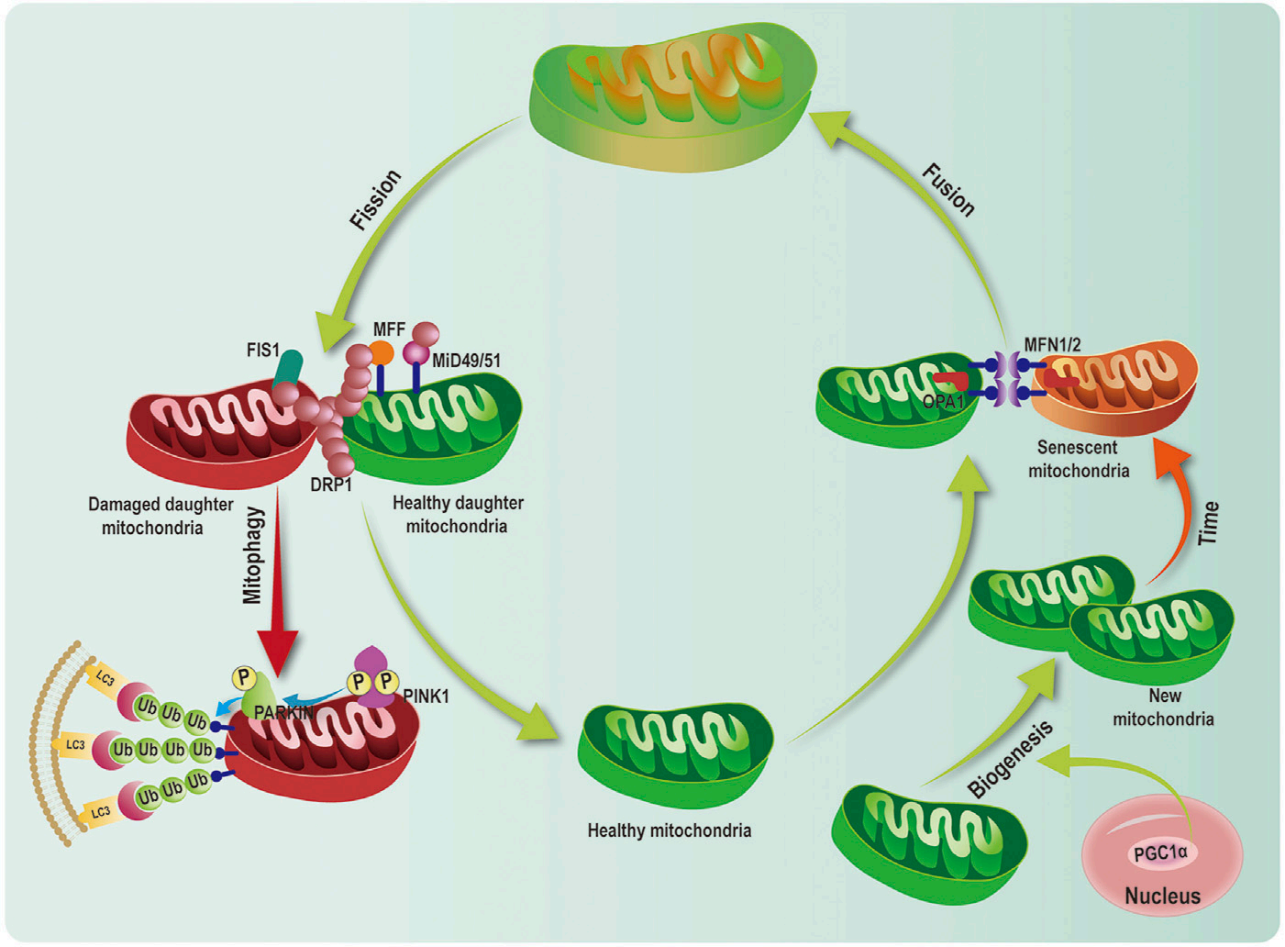
Mitochondrial quality control network. Mitochondrial quality control (MQC) system consists of multiple processes including mitochondrial biogenesis, dynamics (fusion and fission), and mitophagy. Peroxisome proliferator-activated receptor *γ* coactivator 1 alpha (PGC-1*α*), acted as critical transcriptional cofactor, activates mitochondrial biogenesis. Mitofusin 1/2 (MFN1/2) and optic atrophy1(OPA1) regulate mitochondrial fusion. Dynamin-related protein 1 (DRP1) and mitochondrial fission factor (MFF), fission 1(FIS1), mitochondrial dynamics proteins of 49 and 51 kDa (MiD49/51) modulate mitochondrial fission. PINK1 and PARKINpathway is the classical ubiquitination dependent mitophagy pathway, which leads to mitophagy.

Mitochondria are highly dynamic organelles that constantly undergo mitochondrial fusion and division. Fusion and fission are both regulated by members of the dynamin-related protein (DRP) family, including MFN1 and 2, optic atrophy 1 (OPA1), and DRP1. These proteins comprise a large self-assembling GTPases (Praefcke and McMahon, 2004). Fusion of outer mitochondrial membrane (OMM) requires MFN1 and MFN2 to promote fusion of adjacent organelles *via* GTP hydrolysis. In contrast, the fusion of the inner mitochondrial membranes is regulated by the inner membrane protein, OPA1 (Liesa et al., 2009). The mitochondrial fission requires DRP1, which is localized in the cytosol. Upon the recruitment by receptor proteins (mitochondrial fission factor (MFF), fission protein 1(FIS1), mitochondrial dynamics proteins of 49 and 51 kDa (MiD49/51), DRP1 transfers to OMM. DRP1 and receptors form an oligomeric complex that results in constricting to garrote the organelle (Figure 5) (Smirnova et al., 2001).

PGC-1*α* is involved in the regulation of mitochondrial dynamics through the control of expression of core genes. It has been reported that exercise drives the enhancement of expression of MFN1 andMFN2, as well as PGC-1*α* and its coactivators of mitochondrial biogenesis, estrogen-related receptor *α* (Cartoni et al., 2005). Furthermore, the upregulation of MFN1 and MFN2 expression is related to PGC-1*α* in muscle cells. PGC-1*α* stimulated the transcriptional activity of the *Mfn2* promoter, which was mediated by the endogenous ERR*α*, thus indicating that PGC-1*α* and ERR*α* play a synergic role in increasing *Mfn2* mRNA (Soriano et al., 2006). Moreover, MitoQ treatment upregulates MFN2 expression *in vitro*. However, when PGC-1*α* was knockdown by siPGC-1*α*, MFN2 levels did not markedly change treated with MitoQ (Xi et al., 2018). These results demonstrate that PGC-1*α* leads to the transcriptional upregulation of *Mfn2* mediated by MitoQ. Consistent with Vitro results, MFN1, MFN2and DRP1expression are significantly reduced in muscle deletion of PGC-1*α* ^−/−^ in mice compared to wild type (WT) (Zechner et al., 2010). Analysis results of mitochondrial morphology by electron microscopy reveals small, fragmented, and thin mitochondria with largely different in sizes and a reduction in mitochondrial density in PGC-1*α* ^−/-^ mice compared with WT (Zechner et al., 2010). In accordance, recent studies also reveal that mitochondria are higher fragmented in the muscle of PGC-1*α* ^−/-^ young mice compared with young WT (Halling et al., 2017; Halling et al., 2019). Accumulating evidence shows that PGC-1*α* directly interacts with the promoter of the *Drp1* gene to regulate DRP1 levels. DRP1 alteration leads to mitochondrial fission (Ding et al., 2018; Lei et al., 2021). A recent study shows that upregulation of PGC-1*α* can increase expression of MFN2 and OPA1 and decrease expression of DRP1 and FIS1, which mediates the balance fusion and fission (Sui et al., 2021). Together, PGC-1*α* serves as an important modulator of mitochondrial fusion and fission *via* mainly regulating MFN1, MFN2, and DRP1, which keeps mitochondrial network balance.

### PGC-1*α* and Mitophagy

Mitophagy selectively eliminates superfluous and damaged mitochondria to maintain mitochondrial homeostasis (Pickles et al., 2018). Increasing evidence demonstrates that PINK1/PARKIN pathway is the most critical ubiquitination-dependent mitophagy pathway (Youle and Narendra, 2011). Upon depolarization of mitochondrial membrane potential, PINK1 accumulates at the OMM. Furthermore, it recruits the E3 ubiquitin ligase PARKINand phosphorylates PARKIN at Ser65 Lazarou et al., 2015. Activation of PARKINpolyubiquitinates mitochondrial proteins, which are then recognized by autophagy receptors (optineurin (OPTN), p62 [or SQSTM1), NDP52 and neighbor of BRCA1 gene 1(NBR1)] (Lazarou et al., 2015). Then, these complexes bind to Microtubule-associated proteins 1A/1B light chain 3 (LC3) to form the autophagosome, which fuses with the lysosome, resulting in degradation of the mitochondria (Figure 5) (Lazarou et al., 2015). In addition to classical PINK1/PARKIN—related mitophagy, other mitophagy receptors have been reported to involve in mitophagy, including FUN14 domain-containing protein 1(FUNDC1), and Nip3-like protein X (NIX)/BCL-2/adenovirus 19-kd interacting protein 3(BNIP3), AMBRA1, Bcl-2-like protein 13 (Bcl2-L-13), FKBP8, and prohibitin2 (PHB2) (Bhujabal et al., 2017; Fivenson et al., 2017; Wei et al., 2017; Lampert et al., 2019). These receptors can directly interact with LC3-II and induce mitophagy.

Emerging evidence has shown that PGC-1*α* is also an essential element in the regulation of mitophagy. The PGC-1*α* may potentially mediate PINK1 transcriptional activity, which then increases PINK1 levels (Choi et al., 2014; Zhang et al., 2019). PGC-1*α* can also indirectly elevates activation of the PINK1-PARKIN pathway *via* the ERR*α*-SIRT3 pathway, thus mediating the degradation of damaged mitochondria (Ziviani and Whitworth, 2010). Recently, a study shows that PGC-1*α* activates NRF1 to promote mitochondrial biogenesis. Activation of NRF1 also binds to the classic consensus site (‐186/‐176) in the promoter of FUNDC1 to enhance its expression. FUNDC1 interacted with LC3 induces autophagic flux (Liu et al., 2021). Vivo studies demonstrate overexpression of PGC-1*α* in mice elevates autophagy flux. Similarly, it has detected thatthe expression of BNIP3, LC3II, and Beclin1 is upregulated and p62 is downregulated in muscle-specific PGC-1*α* transgenic mice than WT (Lira et al., 2013; Greene et al., 2015). This sign indicates increased basal autophagy flux. Yet another study finds that the downregulation of PGC-1*α* can upregulate BNIP3 in chondrocytes, ultimately inducing clearance of damaged mitochondrial (Kim et al., 2021). This distinct result might attribute to the different genetic background. Taken together, PINK1-Parkin dependent or independent mitophagy pathway is under control by PGC-1*α*.

## The Role of PGC-1*α* in Heart Failure

Mitochondrial functional homeostasis is majorly orchestrated by mitochondrial biogenesis and mitophagy. Heart is a very high energy demand organ, mitochondria occupy ∼40% of adult cardiomyocyte volume and plays a pivital role in the cardiac functions (Zhou and Tian, 2018; Gottlieb et al., 2021). Heart failure caused by various etiologies is characterized by mitochondrial dysfunction, which in turn leads to further cardiac dysfunction. It has been observed PGC-1*α* expression is reduced, accompanied by repression of mitochondrial biogenesis, abnormality of mitochondrial dynamics, impairment of mitophagy and energy defect in patient and animal models of HF (Sebastiani et al., 2007; Goh et al., 2016). Overexpression PGC-1*α* in heart increases normal morphological mitochondria, enhances mitophagy and elevates mitochondrial respiration at 3 months, which maintains cardiac homeostasis at physiological condition (Zhu et al., 2019). These manifest that PGC-1*α*-mediated MQC might play a critical role in HF.

### The Change of PGC-1*α* Expression in Heart Failure

A study found that the protein level of PGC-1*α* was unchanged in heart failure patients compared to normal donors (Hu et al., 2011). However, different study groups detected downregulation of PGC-1*α* in heart and in serum (Garnier et al., 2009; Chen et al., 2019; Joseph, 2019). Xu’s group further found that levels of PGC-1*α* is low along with low left ventricular ejection fraction (LVEF). This indicates that serum PGC-1*α* is associated with left ventricular ejection fraction (LVEF) in patients with HF (Chen et al., 2019). In accordance with heart failure patients, the levels of PGC-1*α* in animal models also is diverse from different studies. Multiple reports illustrate that PGC-1*α* expression is reduced after transverse aortic constriction (TAC), which inhibits the expression of mitochondrial genes and causes important deficiencies in cardiac energy reserves and function (Lehman and Kelly, 2002; Arany et al., 2006; Lu et al., 2010; Piquereau et al., 2017). Yet some studies observed that myocardial PGC-1*α* level was not decreased in the mice following pressure overload-induced heart failure (Hu et al., 2008; Bhat et al., 2019). Recently, Wang and his coworker observed the expression of PGC-1*α* at multiple time points after TAC. The result showed that the expression of PGC-1*α* initially increased 5 days after TAC, but the expression of PGC-1*α* began to reduce 14 days after TAC (Wang et al., 2018). This finding suggests that the expression of PGC-1*α* is fluctuant in the development and progression of HF. This also explains the various outcomes of these different studies regarding PGC-1*α* expression in heart failure. A large body of evidence demonstrates that the mice developed cardiac hypertrophy at 7 days and heart failure at 28 days under overload pressure. PGC-1*α*, as a primary mitochondrial biogenesis regulator, is almost coincident with this condition, indicating that PGC-1*α*-mediated MQC play an important role in the pathogenesis of HF.

### The Effect of PGC-1*α* Deletion in the Heart

Genetic ablation mice are used to explore the function of PGC-1*α* (Table 1). A report from Kelly’s group exhibited normal chamber sizes and ventricular function in deletion of PGC-1*α* in mice at ages 4∼6 months (Leone et al., 2005). In accordance, Chen and colleagues showed that PGC-1*α* ^−/−^ mice did not reveal significant differences in cardiac phenotype and heart ratio under basal conditions compared with WT (Lu et al., 2010). The Spiegelman group displayed that heart structure was normal and mitochondrial biogenesis was not impaired in PGC-1*α* ^−/−^ mice. Whereas, the heart contractile function was significantly deficient in PGC-1*α* lacking mice compared to WT (Arany et al., 2005). Compared with systemic PGC-1*α* knockout mice, cardiac-specific PGC-1*α* knockout mice have more severe impairment in cardiac function. PGC-1*α* reduction represses mitochondrial biogenesis which induces the inhibition of mitophagy further imparing MQC. The mitochondrial content is further alleviated by the accumulation of damaged mitochondria. The Oka group observed that cardiac deletion of PGC-1*α* in mice resulted in enlargement of left ventricular diameters accompanied by cardiac systolic dysfunction (Bhat et al., 2019). Gene expression analyses unveiled increased expression of HF markers atrial natriuretic peptide (ANP), B-type natriuretic peptide (BNP), which suggests PGC-1*α* ^−/−^ mice might develop HF (Bhat et al., 2019). Another study also supports this conclusion and found that ejection fraction (EF%∼29%) significantly depressed and left ventricular (LV) volume increased (Karkkainen et al., 2019). PGC-1*α* deficiency in cardiomyocytes leads to compromised metabolism as well as reduced mitochondrial function. Furthermore, mice lacking PGC-1*α* developed cardiomyopathy at 17 weeks and premature death occurred at 25 weeks (Karkkainen et al., 2019).

**TABLE 1:** Cardiac phenotypes of PGC-1*α* KO and PGC-1*α* overexpression.

Transgenic mouse	Cardiac phenotypes	References
PGC-1*α* KO- systemic	Normal chamber sizes and ventricular function	Leone et al. et al., 2005
PGC-1*α* KO- systemic	Normal cardiac sizes and normal ventricular function and heart ratio	Lu et al. et al., 2010
PGC-1*α* KO- systemic	Normal cardiac size, but significant contractile deficiencies	Arany et al. et al., 2005
PGC-1*α* KO-cardiac specific	Cardiac hypertrophy and cardiac systolic dysfunction	Bhat et al. et al., 2019
PGC-1*α* KO-cardiac specific	Cardiac hypertrophy and decrease of ejection fraction, die at 25 weeks	Karkkainen et al. et al., 2019
PGC-1*α* KI- cardiac specific	Impairment of sarcomeric structure, increase of heart size, decrease of contractile function, die at 6 weeks	Lehman et al. et al., 2000
PGC-1*α* KI-cardiac specific (Tetracycline-inducible)	Mild left ventricular dilatation and depressed ventricular function after induction (4 weeks)	Russell et al. et al., 2004
PGC-1*α* KI—systemic (moderate overexpression)	Normal cardiac function at 3 months	Zhu et al. et al., 2019

Together, these studies indicate that PGC-1*α* deficiency, affects MQC and metabolism, ultimately leads to the development and progression of HF under basal conditions. Moreover, PGC-1*α* ^−/−^ mice develop more profound cardiac dysfunction and clinical heart failure under stressful stimuli such as TAC than WT (Arany et al., 2006; Lu et al., 2010; Bhat et al., 2019). After TAC, PGC-1*α* ^−/−^ mice showed a higher ratio of heart weight to body weight and increase of LV fibrosis compare with sham, which is a sigh of heart failure. Meanwhile contractile performance was aberrant and mortality rate are high. Taken together, PGC-1*α* expression is an essential factor in maintaining normal heart function.

### The Effect of Enhancement of PGC-1*α* in the Heart

PGC-1*α* ^−/-^ mice from different group almost show abnormal cardiac baseline phenotype. Its cardiac function was more worsened in response to pressure overload stimulation than sham. Therefore, it suggests that the enhancement of PGC-1*α* might serve as a therapeutic strategy (Table 1). Cardiomyocyte-specific overexpression of PGC-1*α* contributed to a significant mitochondrial proliferation (Lehman et al., 2000). Uncontrolled mitochondrial proliferation replaced the sarcomeric assembly, which impaired the sarcomeric structure. These transgenic mice also showed increase of heart size, enlargement of four-chamber consistent with a dilated cardiomyopathy and severe decrease of global contractile function. Finally, all transgenic mice died at 6 weeks (Lehman et al., 2000). Another study found that doxycycline (DOX) - induced PGC-1*α* expression in adult mouse hearts also elevated mitochondrial biogenesis, but the mitochondrial ultrastructure appeared abnormal such as vacuoles. (Russell et al., 2004). PGC-1*α* knock in mice occurred cardiac hypertrophy and biventricular dilatation. Echocardiograms revealed repression of ventricular function. These alterations can reverse by removing DOX or cessation of PGC-1*α* overexpression.

These findings manifest that excessive PGC-1*α* expression does not exert a therapeutic role but facilitates the development of heart failure. Recently, several study groups generated a transgenic (TG) mouse model of moderate overexpression of PGC-1*α*(∼3-fold) in the heart, whose cardiac function at baseline was not altered (Karamanlidis et al., 2014; Pereira et al., 2014; Zhu et al., 2019). Moderate PGC-1*α* expression maintained mitochondrial biogenesis, mitophagy and cardiac homeostasis during aging. Nevertheless, a moderate level of PGC-1*α* overexpression did not preserve cardiac function during pressure overload. Directly excessive increase in PGC-1*α* can contribute to various changes, including dramatic enhancement of mitochondrial numbers, enlargement of heart chambers and impairment of cardiac function. Fine-tuning the expression of PGC-1*α* can maintain cardiac homeostasis, but the degree of increase of PGC-1*α* is not sufficient to protect the heart from overload pressure. Thus, it is necessary to consider the dose and period of enhancement of PGC-1*α* so that this strategy can achieve an optimal effect.

## Conclusion

PGC-1*α* is well known as a transcriptional coactivator, which can be involved in maintaining MQC *via* regulation of mitochondrial biogenesis, mitochondrial dynamics, and mitophagy (Puigserver et al., 1998; Zhang et al., 2019). Its activity and expression are crucial for its roles in physiology and pathology conditions. PGC-1*α* is regulated by transcriptional and posttranslational levels. Transcriptional factors like CREB, MEF2C, MEF2D, YY1 can enhance the PGC-1*α* expression, but NF-κB, Hes1 or smad3 can inhibit the PGC-1*α* levels (Handschin et al., 2003; Cunningham et al., 2007; Yadav et al., 2011; Shi et al., 2013; Uittenbogaard and Chiaramello, 2014; Stotland and Gottlieb, 2015; Ruiz-Andres et al., 2016; Han et al., 2017). Its activity can be elevated through the methylation of PRMT1, phosphorylation of MAPK, AMPK, or deacetylation of SIRT1 (Nemoto et al., 2005; Teyssier et al., 2005; Jager et al., 2007; Canto et al., 2009; Tang, 2016; Ye et al., 2019). In contrast, PGC-1*α* can be inhibited by GCN5, AKT, S6 Kinase, GSK3*β* (Gerhart-Hines et al., 2007; Li et al., 2007; Anderson et al., 2008; Kelly et al., 2009; Dominy et al., 2010; Lustig et al., 2011). Heart failure is always associated with mitochondrial dysfunction, thus PGC-1*α*-mediated MQC plays an important role in HF. Increasing evidence displays that the PGC-1*α* level is fluctuated in response to the development of HF (Wang et al., 2018). Indeed, downregulation of PGC-1*α* is a common character at the late stage of HF (Garnier et al., 2009; Lu et al., 2010; Chen et al., 2019; Joseph, 2019). In loss-of-function models, deletion of PGC-1*α* in mice exacerbates cardiac function under pressure overload compared with WT (Arany et al., 2006; Lu et al., 2010; Bhat et al., 2019). These clarify that PGC-1*α* mediates mitochondrial fitness is an important factor in the development and progression of HF. Nevertheless, genetic PGC-1*α* overexpression is not a protective effect, which causes abnormality of mitochondrial ultrastructure and impairment of cardiac function (Lehman et al., 2000; Russell et al., 2004). Moderate overexpression of PGC-1*α* does not change cardiac homeostasis, while It is not sufficient to sustain contractile function upon stressful stimulation (Karamanlidis et al., 2014; Pereira et al., 2014; Zhu et al., 2019). Thus, these approaches suggest that it is important to explore the optimal dose and period of increase in PGC-1*α*, which can achieve the ideal therapeutic effect.

## Glossary

ANP: Atrial Natriuretic Peptide
AMPK: AMP-Activated Protein Kinase
Arg: Arginine
BNP: B-type Natriuretic Peptide
BNIP3: Bcl-2/Adenovirus E1B 19-kd Interacting Protein 3
Bcl2-L-13: Bcl-2-like protein 13
cAMP: Cyclic Adenosine Monophosphate
CaMK: Calcium/Calmodulin-Dependent Protein Kinase
CnA: Calcineurin A
CoA: Coenzyme A
CREB: cAMP-Response Element-Binding Protein
CytC: Cytochrome C
DOX: Doxycycline
DRP1: Dynamin- Related Protein 1
DRP: Dynamin-Related Protein
ETC: Electron Transport Chain
ERR*α*: Estrogen-Related Receptor *α*
FAO: Fatty Acids Oxidation
FAs: Oxidation of Fatty Acids
FIS1: Fission Protein 1
FUNDC1: FUN14 Domain-Containing Protein 1
GCN5: General Control of Amino Acid Synthesis 5
GSK3*β*: Glycogen Synthase Kinase 3beta
HDAC: Histone Deacetylase
HF: Heart Failure
LC3: Microtubule-Associated Proteins 1A/1B light chain 3
LVEF: Left Ventricular Ejection Fraction
LV: Left Ventricular
MAPK: Mitogen-Activated Protein Kinase
MEF2C: Myocyte Enhancer Factors 2C
MEF2D: Myocyte Enhancer Factors 2D
MFF: Mitochondrial Fission Factor
MFN1: Mitofusin1
MFN2: Mitofusin2
MiD49/51: Mitochondrial Dynamics Proteins of 49 and 51 kDa
MQC: Mitochondrial Quality Control
mTOR: Mammalian Target of Rapamycin
mtDNA: Mitochondrial DNA
NAD^+^: Nicotinamide Adenine Dinucleotide
NBR1: Neighbor of BRCA1 Gene 1
NF-κB: Nuclear Factor-κB
NIX: Nip3-like Protein X
NRF1: Nuclear Respiratory Factors 1
NRF2: Nuclear Respiratory Factors 2
OMM: Outer Mitochondrial Membrane
OPTN: Optineurin
OPA1: Optic Atrophy 1
PGC-1*α*: Peroxisome Proliferator-Activated Receptor *γ* Coactivator 1*α*
PGC1*β*: Peroxisome Proliferator-Activated Receptor *γ* Coactivator 1*β*
PHB2: Prohibitin2
PINK1: PTEN-Induced Putative Kinase Protein1
PKA: Protein Kinase A
PPAR*α*: Peroxisome Proliferator-Activated Receptor *α*
PRC: PGC1 related coactivator
PRMT1: Protein Arginine Methyltransferase1
Ser: Serine
SIRT1: Sirtuin1
TAC: Transverse Aortic Constriction
TCA: Tricarboxylic Acid Cycle
TFAM: Mitochondrial Transcription Factor A
TG: Transgenic
TGF*β*: Transforming Growth Factor *β*
Thr: Threonine
WT: Wild type
YY1: Yin Yang 1
